# Sampling Rate of Heart Rate Variability Impacts the Ability to Detect Acidemia in Ovine Fetuses Near-Term

**DOI:** 10.3389/fped.2014.00038

**Published:** 2014-05-05

**Authors:** L. Daniel Durosier, Geoffrey Green, Izmail Batkin, Andrew J. Seely, Michael G. Ross, Bryan S. Richardson, Martin G. Frasch

**Affiliations:** ^1^Department of Obstetrics and Gynecology, CHU Ste-Justine Research Center, Université de Montréal, Montréal, QC, Canada; ^2^Dynamical Analysis Laboratory, Ottawa Hospital Research Institute, University of Ottawa, Ottawa, ON, Canada; ^3^Department of Obstetrics and Gynecology, LA BioMed at Harbor-UCLA Medical Center, Torrance, CA, USA; ^4^Department of Obstetrics and Gynecology, University of Western Ontario, London, ON, Canada; ^5^Faculty of Veterinary Medicine, Animal Reproduction Research Centre (CRRA), Université de Montréal, St-Hyacinthe, QC, Canada

**Keywords:** fetus, fHRV, monitoring, acidosis, asphyxia, hypoxia, sampling rate

## Abstract

**Background:** To evaluate the impact of sampling rate on the predictive capability of continuous fetal heart rate (FHR) variability (fHRV) monitoring for detecting fetal acidemia during labor, we tested the performance of the root mean square of successive differences (RMSSD) in R–R intervals from the ECG when acquired with the sampling rate of 4 Hz currently available in FHR monitors, in comparison to the gold standard of 1000 Hz.

**Methods:** Near-term ovine fetuses (*N* = 9) were chronically prepared with precordial electrodes for recording ECG, vascular catheters for blood sampling, and an umbilical cord occluder. For 1 min every 2.5 min, animals underwent mild partial umbilical cord occlusions (UCO) × 1 h, moderate partial UCO × 1 h, then complete UCO × 2 h, or until arterial pH reached <7.00. Arterial blood samples were drawn at baseline and every 20 min during the UCO series. RMSSD was calculated continuously in 5 min windows using an automated, standardized system (CIMVA.com). Results are presented as mean ± SEM with significance assumed for *p* < 0.05.

**Results:** Repetitive UCO resulted in pH decreasing from 7.35 ± 0.01 to 7.00 ± 0.03. In all nine animals, RMSSD increased from 16.7 ± 1.0 ms at baseline to 44.4 ± 2.3 ms, 70 ± 15 min prior to reaching the pH nadir when sampled at 1000 Hz. When sampled at 4 Hz, RMSSD at baseline measured 36.1 ± 6.0 ms and showed no significant increase during the UCO series until the pH nadir was reached. Consequently, early detection of severe hypoxic-acidemia would have been missed in all fetuses.

**Conclusion:** RMSSD as a measure of fHRV when calculated from FHR sampled at 1000 Hz allowed for the early detection of worsening hypoxic-acidemia in each fetus. However, when calculated at the low sampling rate of 4 Hz used clinically, RMSSD remained unchanged until terminally when the nadir pH was reached. For early detection of fetal acidemia during labor, more sensitive means of acquiring FHR are therefore recommended than currently deployed, e.g., trans-abdominal fetal ECG.

## Introduction

There is an urgent need to identify early signs of fetal acidemia during labor because severe acidemia is associated with increased risk of lasting neurological deficits and current diagnostic procedures are suboptimal (1–3). Variations in fetal vagal activity can be measured by monitoring fetal heart rate (FHR) variability (fHRV) (4, 5). FHR and fHRV are regulated by a complex interplay of the parasympathetic and sympathetic nervous systems accounting for the baseline FHR as well as short-term and long-term fHRV, which show linear and non-linear properties (6). These fHRV properties are differentially affected by fetal acidemia (7–9).

One pivotal measure of fHRV is the RMSSD, the root mean square of the successive differences of R–R intervals in the ECG (10). Changes in RMSSD reflect modulation of fHRV by parasympathetic (vagal) activity on a beat-to-beat time scale and are more precise in capturing vagal activity than the current short-term FHR variation measures used clinically in electronic FHR monitoring (EFM) (11). However, this precision depends on the sampling rate of the ECG signal from which the R–R intervals and the subsequent fHRV are derived (10).

We have shown that RMSSD is a measure of the maturation of the vagal branch of the autonomic nervous system and that RMSSD is reduced by atropine (a cholinergic antagonist) in fetal sheep near-term (5, 7). RMSSD also increases during severe fetal acidemia (pH ~7.09) induced by 4 min umbilical cord occlusions (UCO) 30 min apart in near-term fetal sheep (7). Therefore, RMSSD is a potential marker for worsening acidemia.

In the present study, we aimed at further evaluating the impact of fetal ECG sampling rate on the benefit of continuous fHRV monitoring in predicting fetal acidemia. We tested the performance of RMSSD as a measure of fHRV and change with worsening fetal acidemia when acquired with the sampling rate of 4 Hz currently available in FHR monitors, in comparison to the gold standard of 1000 Hz. Based on evidence from animal and human clinical studies, we propose that longitudinal fHRV monitoring during labor will allow us to improve early diagnosis of fetal acidemia, but this will be impacted by ECG sampling rate.

## Materials and Methods

### Surgical preparation

Nine near-term ovine fetuses [123 ± 2 days gestational age (GA), normal ovine gestation is 145 days] of mixed breed were surgically instrumented. The anesthetic and surgical procedures and postoperative care of the animals have been previously described (3, 12). Briefly, polyvinyl catheters were placed in the right and left brachiocephalic arteries, the cephalic vein, and the amniotic cavity. Stainless steel electrodes were sewn onto the fetal chest to monitor the electrocardiogram (EKG). A polyvinyl catheter was also placed in the maternal femoral vein. In addition, an inflatable silicon rubber cuff (In vivo Metric, Healdsburg, CA, USA) for UCO was placed around the proximal portion of the umbilical cord and secured to the abdominal skin. Antibiotics were administered intravenously to the mother (0.2 g trimethoprim and 1.2 g sulfadoxine, Schering Canada Inc., Pointe-Claire, QC, Canada) and the fetus and into the amniotic cavity (one million IU penicillin G sodium, Pharmaceutical Partners of Canada, Richmond Hill, ON, Canada). Amniotic fluid lost during surgery was replaced with warm saline. The uterus and abdominal wall incisions were sutured in layers and the catheters exteriorized through the maternal flank and secured to the back of the ewe in a plastic pouch.

Postoperatively, animals were allowed 4 days to recover prior to experimentation and daily antibiotic administration was continued. Arterial blood was sampled for evaluation of maternal and fetal condition and catheters were flushed with heparinized saline to maintain patency. Animals were 129 ± 1 days GA on the first day of experimental study. Animal care followed the guidelines of the Canadian Council on Animal Care and was approved by the University of Western Ontario Council on Animal Care.

### Experimental procedure

Fetal animals were studied over a ~6-h period. After a 1–2 h baseline control period, all animals underwent mild, moderate, and severe series of repetitive UCOs by graduated inflation of the occluder cuff with a saline solution. During the first hour following the baseline period, mild variable FHR decelerations were elicited with a partial UCO for 1 min duration every 2.5 min, with the goal of decreasing FHR by ~30 bpm, corresponding to an ~50% reduction in umbilical blood flow (13, 14). During the second hour, moderate variable FHR decelerations were elicited with increased partial UCO for 1 min duration every 2.5 min with the goal of decreasing FHR by ~60 bpm, corresponding to a ~75% reduction in umbilical blood flow (14). Animals then underwent severe variable FHR decelerations with complete UCO for 1 min duration every 2.5 min until the targeted fetal arterial pH of <7.0 was detected or 2 h of severe UCO had been carried out, at which point the repetitive UCOs were terminated. All animals were then allowed to recover for 48 h following the last UCO. Fetal arterial blood samples were drawn at baseline, at the end of the first UCO of each series (mild, moderate, severe), and at 20 min intervals (between UCOs) throughout each of the series, as well as at 1, 24, and 48 h of post-insult recovery. For each UCO series blood gas sample and the 24 h recovery sample, 0.7 ml of fetal blood was withdrawn, while 4 ml of fetal blood was withdrawn at baseline, at pH nadir <7.00, and at 1 and 48 h of recovery. The amounts of blood withdrawn were documented for each fetus and replaced with an equivalent volume of maternal blood at the end of day 1 of study.

All blood samples were analyzed for blood gas values, pH, glucose, and lactate with an ABL-725 blood gas analyzer (Radiometer Medical, Copenhagen, Denmark) with temperature corrected to 39.0°C. Plasma from the 4 ml blood samples was frozen and stored for cytokine analysis, and will be reported separately.

After the 48 h recovery blood sample, the ewe and the fetus were killed by an overdose of barbiturate (30 mg sodium pentobarbital IV, MTC Pharmaceuticals, Cambridge, ON, Canada). A post mortem was carried out during which fetal sex and weight were determined and the location and function of the umbilical occluder were confirmed.

### Data acquisition and analysis

A computerized data acquisition system was used to record fetal arterial and amniotic pressures and ECG, which were monitored continuously throughout the baseline and UCO series (15). Arterial and amniotic pressures were measured using Statham pressure transducers (P23 ID; Gould Inc., Oxnard, CA, USA). Arterial blood pressure (ABP) was determined as the difference between instantaneous values of arterial and amniotic pressures. A PowerLab system was used for data acquisition and analysis (Chart 7 for Windows, AD Instruments Pty Ltd., Castle Hill, NSW, Australia).

Pressures and ECG were recorded and digitized at 1000 Hz for further study. For ECG, a 60 Hz notch filter was applied. FHR was additionally triggered and calculated in real time from arterial pressure systolic peaks.

Averaged values of FHR and ABP were calculated from artifact-free recordings of 1 h of baseline, between and during each consecutive variable FHR deceleration induced by UCO (mild, moderate, severe), as reported (16).

**Figure 1:**
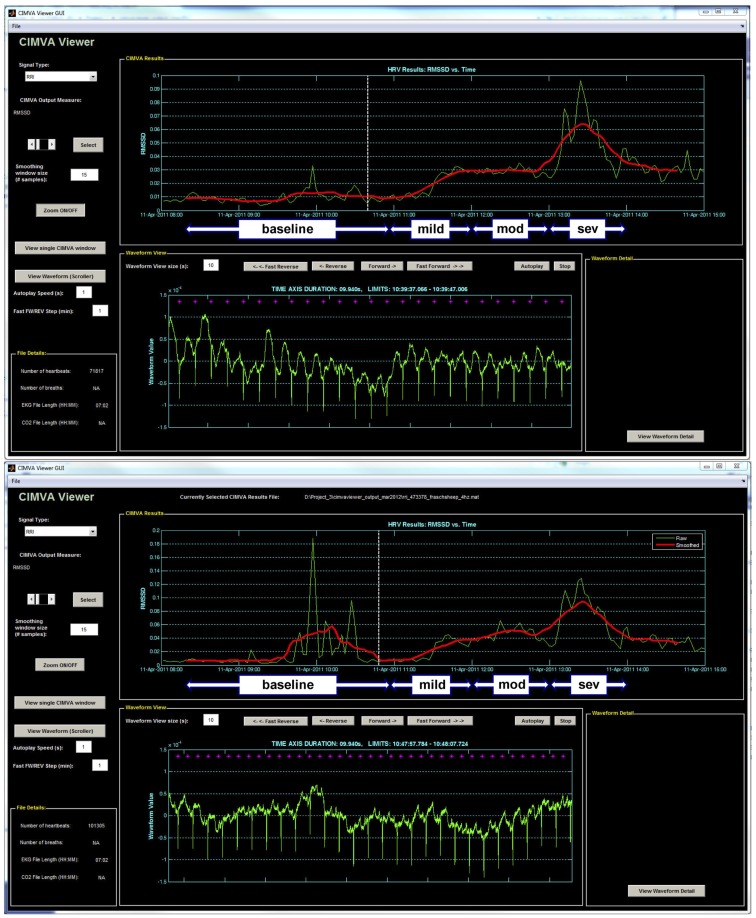
**A representative example of the CIMVA fHRV analysis of RMSSD at (top) 1000 Hz and (bottom) 4 Hz**. Note the gradual rise of RMSSD with worsening acidemia (pH decreasing) and an overestimation of RMSSD at baseline in 4 Hz sampled fHRV. Mild, mod and sev = mild, moderate, and severe UCO series, respectively.

R–R intervals sequence was originally generated from ECG sampled at 1000 Hz. For simulation of the 4 Hz sampling rate, the R–R intervals sequence was interpolated at 4 Hz. RMSSD was calculated from the 1000 and 4 Hz sampled R–R intervals continuously in 5 min windows overlapping by 2.5 min using an automated and standardized CIMVA.com system (Figure 1). Next, average RMSSD values were determined for the 1 h baseline period and each 20 min interval from the UCO experimental period and correlated to the time-matched pH, lactate, and base excess (BE) values. Accordingly, each 20 min interval included ~8 UCO-induced FHR decelerations and the intervening recovery periods.

### Statistical analysis

Normal data distribution was tested using the Kolmogorov–Smirnov test followed by parametric or non-parametric tests, as appropriate. The pH measurements and the temporal change in RMSSD values versus baseline for the 1000 and 4 Hz sampling rates were compared by one-way repeated measures ANOVA followed by Holm–Sidak or one-way repeated measures ANOVA on ranks (Friedman) followed by correction for multiple comparisons (Dunn’s method) as applicable.

All values are expressed as means ± SEM with statistical significance assumed for *p* < 0.05. Pearson or Spearman correlation analysis was performed as appropriate, and *R* values are presented where *p* < 0.05 (SPSS 19; IBM, Armonk, NY, USA).

## Results

Baseline fetal arterial pH (7.35 ± 0.01) as well as FHR (159 ± 5 bpm) and ABP (44 ± 2 mmHg) were within physiological range.

Repetitive fetal UCO resulted in development of marked acidosis with arterial pH decreasing from 7.35 ± 0.01 to 7.00 ± 0.03 and BE from 1.6 ± 0.7 to −13.6 ± 1.1 mEq/l by the end of the UCO study (both *p* < 0.01). ABP increased on average to 75 ± 3 mm Hg during each UCO versus 54 ± 2 mm Hg between each UCO (*p* < 0.05). FHR deceleration depth averaged 66 ± 6 bpm decreasing to 94 ± 6 bpm during each UCO versus 159 ± 3 bpm between each UCO (*p* < 0.05).

**Figure 2:**
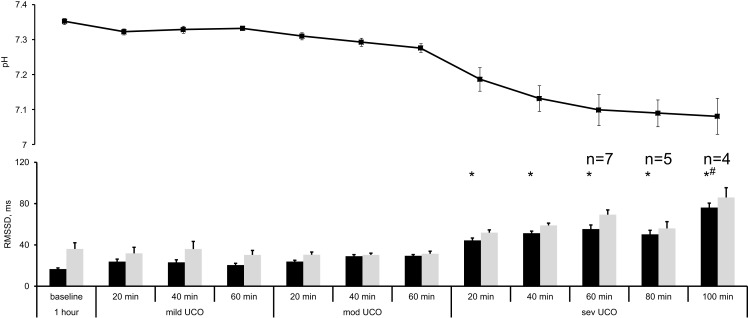
**FHRV analysis of RMSSD changes during worsening acidemia (black) at 1000 Hz and (gray) 4 Hz sampling rates**. Animals reached pH nadir <7.00 between the time points “severe UCO 40 min” and “severe UCO 100 min.” Accordingly, sample sizes of these time points were lower where indicated. For other time points, *N* = 9. Mean ± SEM. *p < 0.05 versus baseline for 1000 Hz sampled fHRV; ^#^ versus baseline for 4 Hz sampled fHRV.

The changes in RMSSD with the repetitive UCO are summarized in Figure 2. When sampled at 1000 Hz, baseline RMSSD measured 16.7 ± 1.0 ms with all animals, then increased to 44.4 ± 2.3 ms during the repetitive UCO 70 ± 15 min prior to reaching the pH nadir (*p* < 0.01). This increase was evident 20 min into severe UCO series. All subsequent measurements of RMSSD until the UCO were stopped showed similar increases compared to baseline RMSSD values (Figure 2, all *p* < 0.01).

When sampled at 4 Hz, baseline RMSSD was found to measure 36.1 ± 6.0 ms. Albeit higher on average, this value was not statistically significantly different from the 1000 Hz sampled baseline measurement (Wilcoxon signed rank *p* = 0.3). During the repetitive UCO, RMSSD showed no definite increase in five of the nine fetuses. In the remaining four fetuses an increase was observed, but only at the time of reaching the pH nadir. Moreover, their RMSSD increase occurred 100 min into the severe UCO series with RMSSD measuring 85.9 ± 9.5 ms (*p* < 0.01) (Figure 2).

## Discussion

In the present study, RMSSD increased early with worsening hypoxic-acidemia in each fetus when sampled at 1000 Hz. This is in line with previous observations in fetal sheep near-term undergoing repetitive UCO and giving rise to worsening hypoxic-acidemia (7). However, when fetal ECG was instead sampled at 4 Hz simulating the low sampling rate for fHRV used clinically in electronic FHR monitors, RMSSD was not significantly changed in five of the fetal sheep and only increased terminally with severe hypoxic-acidemia in the other four animals. The methodological reason for this finding is overestimation of fHRV due to undersampling (17, 18). As pointed out by Merri et al., this overestimation may be further impacted by the degree of the biological variability itself introducing an additional confounder in the fHRV measures derived from low sampled FHR data.

There are several reasons why FHR monitoring fails to detect fetal hypoxic-acidemia as presently used clinically. First, limited information can be derived by visual analysis of the FHR pattern and uterine contractions. Second, present technology for the detection of fetal heart bioelectric events is limited by poor recording of the true bioelectric signal. Trans-abdominal Doppler probes operate on averaged biophysical signals, and scalp electrodes, either filtered or sampled at low frequency, detect a robust but smoothed QRS signal (19). This results in loss of the information embedded in fHRV (5, 7). The time scale of subtle fHRV events requires the temporal resolution of *R* peak detection in the QRS complex within <1 ms (6, 20, 21). Our approach and findings in the fetal sheep model are echoed and supported by the recently published prospective study of human fHRV (18). The commercial FHR acquisition software of a conventional cardiotocogram monitor used in that study sampled FHR derived from the Doppler signal every 2.5 s. The researchers modified this algorithm to read the FHR every 0.5 s (corresponding to 2 Hz, still twice as low as the sampling rate simulated in the present study), thus somewhat increasing the precision of fHRV estimation. Despite the still very low fHRV sampling rate, the linear and non-linear fHRV measures determined from this data set allowed the authors to detect differences between the fHRV of the intrauterine growth-restricted fetuses (IUGR) compared to the healthy fetuses. Notably, the authors reported no difference in the high frequency power spectrum estimates of fHRV between the groups. This is consistent with the main finding of this study that low HRV sampling rates likely overestimate HRV, thus obscuring the true change in HRV due to a pathophysiological state.

Thus, for earlier detection of fetal acidemia during labor as herein shown with RMSSD as a measure of fHRV, more sensitive means of acquiring FHR are recommended than currently deployed in EFM. Candidates for alternative means of fetal assessment for detecting the onset of hypoxic-acidemia include trans-abdominal ECG, already used at the bedside in some jurisdictions, or experimental approaches involving fetal EEG during labor (22–24). Fetal scalp ECG could also be sampled at 1000 Hz, thus permitting true fHRV analyses such as presented here.

Limitations of this study are its small sample size and focus on one particular fHRV measure reflecting vagal modulations of fHRV, the RMSSD. Larger prospective studies in fetal sheep model and human clinical prospective trials of FHR monitoring during labor are needed to provide high resolution R–R data sets with correlated clinical outcomes such as pH and BE at birth. Such data sets could then be studied with a more encompassing panel of fHRV measures to validate their usefulness in intrapartum FHR monitoring for early detection of fetal acidemia. First steps in this direction have been made with respect to sample size, but not yet with respect to the R–R temporal resolution, i.e., sampling rate (25).

### Significance and novelty

Continuous beat-to-beat fHRV monitoring capable of detecting vagal activation due to fetal acidemia may be a useful tool for early detection of hypoxic-acidemia near term.

## Presentation Information

Presented in part at Society for Gynecologic Investigation Annual Meeting 2012.

## Conflict of Interest Statement

The authors declare that the research was conducted in the absence of any commercial or financial relationships that could be construed as a potential conflict of interest.
